# Association between depression and HIV treatment outcomes in a US military population with HIV infection

**DOI:** 10.1186/s12981-021-00350-2

**Published:** 2021-05-12

**Authors:** Brandon Carney, Colton Daniels, Xiaohe Xu, Thankam Sunil, Anuradha Ganesan, Jason M. Blaylock, Karl C. Kronmann, Christina Schofield, Tahaniyat Lalani, Brian Agan, Jason F. Okulicz

**Affiliations:** 1 Brooke Army Medical Center, 3551 Roger Brooke Drive, Fort Sam Houston, TX 78234-6200 USA; 2 Department of Sociology, University of Texas at San Antonio, San Antonio, TX USA; 3 Institute for Health Disparities Research, University of Texas At San Antonio, San Antonio, TX USA; 4 Infectious Disease Clinical Research Program, Department of Preventive Medicine, Uniformed Services University of the Health Sciences, Bethesda, MD USA; 5 Walter Reed National Military Medical Center, Bethesda, MD USA; 6 Naval Medical Center, Portsmouth, VA USA; 7 Henry M. Jackson Foundation for the Advancement of Military Medicine, Inc, Bethesda, MD USA; 8 Madigan Army Medical Center, Joint Base Lewis-McChord, WA USA

**Keywords:** HIV, Depression, Adherence, Antiretroviral therapy, Viral load suppression

## Abstract

**Background:**

Depression is common among HIV-infected individuals and may contribute to suboptimal adherence to antiretroviral therapy (ART) and subsequent inability to attain viral load (VL) suppression. We evaluated associations between depression, self-reported adherence, and longitudinal HIV treatment outcomes in US Military HIV Natural History Study (NHS) participants with and without depression.

**Methods:**

Male NHS participants with available ICD-9 data for mental health diagnoses, Center for Epidemiological Studies Depression (CES-D) measures, and self-reported adherence (SRA) were included. ART use was defined as ART initiation between 2006 and 2010, with follow-up through 2015. SRA was defined as taking 95% of ART doses and continuous ART was defined as longitudinal ART use with gaps  < 30 days. Continuous VL suppression was defined as maintaining VLs  < 200 c/mL on ART. To analyse the association between depression and HIV treatment outcomes, latent class analysis was used to create classes of depression trajectories: low depression (LD), recent onset depression (ROD) and high Depression (HD).

**Results:**

Participants had a mean age of 32 (± 8.3) years at HIV diagnosis, and similar proportions were Caucasian (44.3%) or African American (40.8%). Overall, older participants at HIV diagnosis had greater odds of having 95% self-reported adherence (OR 1.06, 95% CI 1.02–1.12), and African Americans had lower odds (OR 0.41, 95% CI 0.22–0.76) compared to Caucasians (OR 1.49, 95% CI 0.52–4.28). However, there was no difference in SRA by depression trajectory. Participants with HD had an increased odds of taking ART continuously (OR 1.75, 95% CI 0.99–3.09), and those with ROD had significantly higher odds of virologic failure (OR 0.58, 95% CI 0.38–0.91).

**Conclusions:**

Although there was no observed association between depression and SRA, participants with ROD had lower odds of attaining the HIV treatment goal of VL suppression. Continued efforts to identify and aggressively manage mental health disorders is important to success along the HIV care continuum.

## Introduction

Adherence to antiretroviral therapy (ART) is essential for the achievement and long-term maintenance of viral suppression in people living with HIV (PLWH). Durable viral suppression has many benefits including immune reconstitution, reduced risk of AIDS and prevention of onward transmission to sex partners [1–4]. However, concurrent mental health diagnoses such as depression may negatively influence adherence to ART, resulting in failed viral suppression. Major depressive disorder has been shown to be more prevalent in PLWH compared to those without HIV infection [5, 6]. A previous study reported that treatment-naïve patients with mental health disorders had slower rates of virologic suppression after ART initiation [7].

The potential impact of depression on ART adherence has been evaluated in previous studies. For example, one study performed cross-sectional assessments of adherence and demonstrated greater odds of  < 80% ART adherence among HIV infected individuals diagnosed with depression compared to those without depression [8].

Another cross-sectional analysis from 2011 to 2014 in Chicago evaluated multiple psychosocial conditions to include depression and anxiety and their relation to adherence (described as “syndemic”). Controlling for treatment factors and demographics, symptoms of depression were present in 38% of participants and both ART adherence and VL suppression decreased with increasing number of syndemic conditions. While interventions to address these co-morbid psychosocial conditions have been recommended [9–12], there is limited understanding of the specific relationship between clinical depression and ART adherence. Furthermore, the cross-sectional design of these prior studies limits the ability to substantiate links between treatment adherence/viral load suppression and depression among HIV infected persons.

We previously analyzed the relationship between longitudinal depression trajectories and sexual risk behaviors in US Military HIV Natural History Study (NHS), observing that participants with depression were less likely to use condoms and more likely to have sex with multiple partners [13]. The current analysis used the same longitudinal depression trajectories to further understand the potential impact of depression on self-reported adherence and longitudinal HIV treatment outcomes.

## Methods

The NHS is a prospective observational cohort of over 6000 HIV-infected active duty military personnel and beneficiaries. Participants are evaluated approximately every 6–12 months with data collected for demographics, surveys, medical diagnoses, laboratory monitoring, and HIV treatment outcomes. All participants were at least 18 years of age and provided written informed consent for this IRB-approved study. Male NHS participants were included who met criteria for longitudinal mental health diagnosis and survey data, self-reported adherence (SRA) and ART use (n = 549). ART use was defined as ART initiation between 2006 and 2010, with follow-up through 2015. SRA was defined as taking 95% of ART doses within the past month for adherence assessments conducted each study visit and continuous ART was defined as longitudinal ART use with gaps < 30 days. Continuous VL suppression was defined as maintaining VLs  < 200 c/mL on ART for all VL assessments over the same time period.

Self-reported Center for Epidemiological Studies Depression (CES-D) measures standardized by the American Psychological Association [14] and ICD-9 codes for depression collected from 2006 to 2010 were evaluated with self-reported depressive symptoms from 2014 to 2015 as previously described [13]. Using a cutoff score of 16 to identify respondents at risk for clinical depression, the 20-item CES-D scores from 2006 (time period 1), 2007–08 (time period 2), and 2009 (time period 3), respectively, were coded such that 1 represented 16 or higher (at risk for clinical depression) and 0 indicating below 16 (less risk). The cutoff value of 16 was based on recommendations designated by the American Psychological Association (APA). For the self-reported days of depression, participants were asked at study visits, “Thinking about your mental health, which includes stress, depression, and problems with emotions, for how many days during the past 30 days was your mental health not good?” Responses were coded into 1 being 1 or more days (at risk for clinical depression) and 0 indicating 0 days (no risk). These measures represent time periods four and five (2014–2015).

To utilize these self-reported depressive symptoms over the five time periods, Latent class analysis (LCA) was conducted to identify and categorize unmeasured, longitudinal depression trajectories rather than static group membership. LCA results revealed three trajectories: high depression (HD; ICD-9 diagnosis for depression or CES-D score of one indicating higher risk of depression), recent onset depression (ROD; depressive symptoms present in periods four and five only), and low depression (LD; CES-D score of 0 indicated less depression risk). Socio- demographic characteristics were used as control measures. The three depression trajectories were further dummy-coded with LD serving as the reference for multivariable statistical modeling. The covariates included in the adjusted analyses were age at HIV diagnosis, race/ethnicity, and CD4 count at the time of HIV diagnosis. The LCA was performed using MPlus and all other statistical analyses were conducted using SPSS.

## Results

The mean age at HIV diagnosis for the 549 participants included in this study was 32 years, and similar proportions were Caucasian (44%) and African American (41%), with those in the Other category representing a smaller portion of the sample (15%; Table 1. Participants had a mean CD4 count at HIV diagnosis of 502 ( ± 246) cells/µL and mean VL of 4.3 log10 (± 0.9) copies/mL. For depression outcomes, 90% had a high risk CES-D score (≥ 16) as opposed to having a coded diagnosis of depression. LCA for the CES-D and self-reported depressive symptoms categorized participants as LD (n = 326, 59.4%), ROD (n = 137, 25%), and HD (n = 86, 15.7%).

**Table 1:** Characteristics of NHS participants

Characteristic	Number (n) or Mean (± SD)
Number, n	549
Age	52.1 (10)
Mean age at HIV diagnosis (years)	32.7 (8.3)
Mean CD4 count at HIV diagnosis (cells/µL)	502.8 (246.1)
Mean viral load at HIV diagnosis (log10copies/mL)	4.3 (0.9)
Mean viral load at ART (log10copies/mL)	4.3 (0.9)
Time from HIV diagnosis to ART start (years)	5.3 (4.8)
Mean time from HIV negative to seroconversion (years)	2.0 (1.9)
Independent variables	
Depression classes	
Low depression throughout (reference)	326
Recent onset depression	137
High depression throughout	86
Race	
Caucasian	243
African-American	224
Other	82

Some patients did not have information in their medical records on CD4 cell count at HIV diagnosis (9%) or VL at ART start (8%)

Overall, participants with increased age at HIV diagnosis (per 1 year) had higher odds of having 95% SRA (OR 1.06, 95% CI 1.02–1.12) while African Americans, compared to Caucasians, had significantly lower odds in our study (OR 0.41, 95% CI 0.22–0.76) Table 2. Individuals with ROD or HD did not demonstrate significant differences in SRA.

**Table 2:** Logistic regression analyses of depression on HIV clinical behaviors and reports

Variables	OR	95% CI	P-value
Self-reported adherence (≥ 95% of ART doses)			
Age at HIV diagnosis (per 1 year increase)	0.97	0.97–0.99	0.017
Race (Caucasian = reference)			0.273
African American	1.28	0.82–2.00	0.273
Other	0.69	0.36–1.33	0.385
Recent onset depression	0.92	0.56–1.49	0.818
High depression	1.17	0.66–2.01	0.603
CD4 count at HIV diagnosis ≥ 500 cells/uL	5.22	3.45–7.89	0.001
Delay from HIV diagnosis to ART start			
Age at HIV diagnosis	0.97	0.97–0.99	0.017
Race (Caucasian = reference)			0.0237
African American	1.28	0.82–2.00	0.237
Other	0.69	0.36–1.33	0.385
Recent onset depression	0.92	0.56–1.49	0.818
High depression	1.17	0.66–2.01	0.603
CD4 count at HIV diagnosis ≥ 500	5.22	3.45–7.89	0.001
Continuous ART (break < 30 days)			
Age at HIV diagnosis	0.99	0.96–1.01	0.218
Race (Caucasian = reference)			0.671
African American	1	0.67–1.51	0.988
Other	1.29	0.72–2.31	0.401
Recent onset depression	1.28	0.82–2	0.272
High depression	1.75	0.99–3.09	0.057
CD4 count at HIV diagnosis ≥ 500	1.07	0.73–1.57	0.726
Continuous viral load suppression (< 200 c/mL)			
Age at HIV diagnosis	1.01	0.99–1.03	0.451
Race (Caucasian = reference)			0.43
African American	0.92	0.6–1.4	0.693
Other	1.37	0.74–2.53	0.312
Recent onset depression	0.58	0.38–0.91	0.017
High depression	1.15	0.65–2.06	0.63
CD4 count at HIV diagnosis ≥ 500	1.15	0.77–1.71	0.495

LD is used as reference group

Participants with a CD4 count  ≥ 500 cells/L at HIV diagnosis had higher odds of delay from HIV diagnosis to starting ART (OR 5.22, 95% CI 3.45–7.89). This finding is likely due to the CD4 count thresholds from which to start therapy based on existing guidelines at that point in time. Additionally, increased age at HIV diagnosis (per 1 year) had lower odds of delay in starting ART (OR 0.97, 95% CI 0.97–0.99). No significant association was found with race for delay in starting ART.

Those with HD demonstrated an increased odds of taking ART continuously (OR 1.75, 95% CI 0.99–3.09), but not those with ROD (OR 1.28, 95% CI 0.82–2.0). Other variables did not show statistical significance for continuous ART. Participants with ROD (0.58, 95% CI 0.38–0.91) had significantly lower odds of continuous VL suppression whereas no difference was observed for HD participants (OR 1.15, 95% CI 0.65–2.06).

## Discussion

Analysis of different variables to include ART adherence and VL suppression among HIV-infected persons with a coded diagnosis of depression or self-reported data from the NHS cohort demonstrated that mental health comorbidities may influence these important elements of longitudinal HIV disease management. Historically, for ART to be most effective in preventing HIV virologic failure, ART adherence of 95% or greater is strongly recommended [15] as was investigated for our current study. While the necessary adherence rate for sustained VL suppression likely varies among different classes and combinations of agents included in ART regimens, a high adherence goal is necessary given the intricacies of some ART regimens and the increased morbidity/mortality seen with suboptimal adherence [16]. In our study, increased age at HIV diagnosis per 1 year was associated with better adherence to ART. This reporting of age as a determinant for adherence has been published elsewhere, citing better medication adherence in older individuals (> 35 years) compared with younger patients [17]. Lower age as an independent risk factor for virologic failure has also been appreciated within our NHS cohort in prior literature looking at persistent low-level viremia and virologic failure [18]. Lower adherence in younger individuals may be related to more significant socioeconomic conditions as compared to older individuals or having less experience in interacting with the health care system [19].

Lower adherence was observed in African Americans in our study. Young African American males may be less likely than Caucasian individuals to receive adequate mental health care secondary to economic, social or demographic barriers [20]. Such perceived barriers may result in higher levels of healthcare mistrust than other racial groups, especially in the setting of concurrent psychosocial stressors as previously studied in this specific population [21]. Both measured and unmeasured factors, such as substance use, fear of assigned stigma, and education, may contribute to differences in adherence and warrants further study in our cohort [22].

Another variable assessed was documented adherence to ART over time. Participants in our study having HD had an increased odds of taking ART continuously which differed from other reports [23]. This may be due to overestimation of adherence by participants. While this result wasn’t statistically significant for our cohort and may seem contrary to other study findings, such an associations has been discussed in previous literature. In a cross-sectional prospective survey among 300 HIV-infected adults on ART in Botswana, an 87-question survey to include graded questions about depression was explored in relation to adherence (European Quality of Life instrument). Of these participants, 21% had “severe depression,” and while depression was found to be a predictor of poor adherence rates (p < 0.02), adherence rates were poorest among those just starting ART, most notably within 1–6 months of starting compared to those on ART for  > 12 months [24]. This may be related to side effects or other factors not related to depression. More notably, a meta-analysis looking into depression and HIV treatment interventions found that greater improvements in adherence were found in individuals with more severe depression, using instruments to include CES-D scale measures that were used in our study. It is possible those with classification of more severe depression may perceive a greater opportunity for improvement in both HIV and depression management [25]. Additional studies are warranted to detect associations between SRA and depression categories.

Additionally, participants with ROD had significantly lower odds of achieving viral load suppression compared to those with HD. Many studies have evaluated the effect of depression on virologic response to ART with significant associations identified, even after controlling for adherence [26]. In a study of 198 ART-naïve patients initiating therapy for whom a previously validated model to predict psychiatric illness was employed, patients with such a projected diagnosis were slower to reach VL suppression (AHR 1.2, CI 1.06–1.4), a result that may be attributable to adherence or loss of follow up [7].

One limitation of this study is that all types of mental health data were not available throughout the study period and only a subgroup of the entire NHS cohort was included. The particular diction within ICD-9 codes apropos distinguishing recurrent depression versus Single episode depression for diagnosis is not always established. Another limitation is SRA as this is subject to social desirability bias. Additionally, our findings may not be generalizable to women as only males were included in this analysis. Furthermore, it would be reasonable to incorporate the number of participants undergoing active treatment for depression into future studies as this variable was not included in our analysis.

The relationship between depression and medication adherence among HIV-infected people to attain viral load suppression is complex. The marked improvements in ART have afforded PLWH an opportunity to live significantly longer and healthier lives than ever before. However, such benefits of ART depend on an individual’s ability to adhere to daily medications, which can pose a challenge in those with comorbid mental health diagnoses such as depression. Improved identification and management of concurrent mood disorders are very important in optimizing ART adherence among HIV-infected persons.

## Data Availability

Data for this study are available from the Infectious Disease Clinical Research Program (IDCRP), headquartered at the Uniformed Services University of the Health Sciences (USU), Department of Preventive Medicine and Biostatistics. The Informed Consent Document under which the HIV Natural History Study data were collected specifies that each use of the data will be reviewed by the Institutional Review Board. Furthermore, the data set may include Military Health System data collected under a Data Assurance Agreement that requires accounting for uses of the data. Data requests may be sent to: Address: 6270A Rockledge Drive, Suite 250, Bethesda, MD 20,817. Email: contactus@idcrp.org.
